# Acute Lobar Pneumonia with Cardiac Failure

**Published:** 1882-05

**Authors:** Charles W. Pilgrim

**Affiliations:** Late House Physician, Bellevue Hospital, New York


### CLINICAL REPORTS.

### ARTICLE II.

### ACUTE LOBAR PNEUMONIA WITH CARDIAC FAILURE.

BY CHARLES W. PILGRIM, M.D.,
Late House Physician, Bellevue Hospital, New York.

The patient, a female, about twenty-one years of age, was admitted into ward 22, Bellevue Hospital, at 5:30 P.M., Oct. 13th, 1881. She was in a delirious condition, and could give no history of her sickness. An examination gave the following results : Temperature in axilla, 103° F. ; pulse, 130 per minute, small, feeble, and at times intermittent ; respirations greatly accelerated ; cheeks dusky ; slight cough and dulness and bronchial breathing over the region corresponding to the lower lobe of the left lung. The presence of bronchophony and increase of vocal fremitus could not be ascertained, as the patient was not in a condition to aid in eliciting the signs. The urine contained a trace of albumen. Whiskey, quinine, and carbonate of ammonia were ordered. Ten minims of tincture of digitalis were also given hypodermically. Her condition kept growing worse from hour to hour until midnight, when she died. The autopsy revealed the following conditions of the internal organs :

*Heart.*—A dense *ante mortem* clot in right ventricle. Valves normal.

*Lungs.*—Pleuritic adhesions at the base and posterior portion of each. The lower lobe of left in a state of red hepatization, and the upper one somewhat œdematous. The right also œdematous, but otherwise normal.

*Liver.*—Increased in size and much softer than normal. Color unchanged.

*Kidneys.*—" Cloudy swelling" well marked, the cortex being much thicker and darker than normal. Capsule non-adherent, and surface smooth and vascular.

*Spleen.*—Very soft, and enlarged to at least three times its natural size.

There is nothing very peculiar about the above case, and it is only reported because it is thought that the enlarged and softened spleen and liver, together with the " cloudy swelling" of the kidneys, may be considered evidence in favor of the theory that pneumonia is an essential fever with a local manifestation, the inflammatory process of the lungs bearing the same relation to the febrile disease that the inflammation of the intestinal glands bears to typhoid fever. This doctrine is advocated especially by Juergensen and Flint, but is not yet generally accepted by medical writers and practitioners. Prof. Flint's grounds, briefly stated, for his belief are as follows : " In relation to the morbid anatomy, the quantity of exudation, amounting to from one to two pounds if a single lobe be affected, and sometimes reaching four pounds if the affection embrace an entire lung ; the derivation of this matter from the blood in the branches of the pulmonary artery ; the removal of the exudation by absorption, leaving the air-vesicles intact ; the extension over a lobe by degrees, the progress often being slow ; the invasion successively of a second and third lobe in a certain proportion of cases, and the laws of the disease, as regards the greater liability of the lower lobes, are points which, to say the least, are suggestive of dependence on a constitutional morbid condition, the latter being essentially the disease.

" 2d. Etiology furnishes support of the doctrine in two points of view, namely : First, the local affection is never produced by local causes, such as contusions and penetrating wounds of the chest, nor does it ever occur as a sequence of acute pleurisy ; and, second, all the knowledge which we at present have of the causation is in favor of the primary action of the cause or causes being constitutional. Proof is also afforded by the prevalence of the disease at certain seasons of the year, namely, the vernal months, in this climate, and by the fact that it is more prevalent in some climates than in others. In our own country it is vastly more frequent in the Southern than in the Northern States, and in certain situations in the South, it has been known to prevail to an extent entitling it to be called endemic.

"3d. Passing to the clinical history, the grounds are hardly less substantial than those furnished by the etiology of the disease. The chill, which is usually the first symptomatic event, is more pronounced than in the history of any local affection. It is often as marked as in the cold stage of a paroxysm of intermittent fever. The fever which follows quickly rises, and often in a few hours becomes intense. It is not uncommon for the temperature of the body to be five or six degrees above the normal limit in from four to twelve hours after the attack. Now, this cannot be a symptomatic fever, for within these periods, and often for two or three days, the pneumonic inflammation is so limited as not to furnish the distinctive and easily determined physical signs of the local affection. *As in other fevers, enlargement of the spleen and albuminuria are frequent in this disease* (italics mine). As in typhoid fever defervescence is not determined by conditions which relate to the local affection. Defervescence sometimes begins and ends within twelve hours, or even less, and during this time the physical signs may show that no very marked change has taken place in the pulmonary organs. The disease, like typhoid fever, ends from self-limitation—that is, it ends when the disease has finished its career. It is not uncommon for the career of the fever to end when there is much to be done in the way of resolution before the restoration of the normal pulmonary condition is complete."

The preceding arguments, I think, will meet with the approval of all who have seen much of this disease. It is also well known that the influence of large doses of sulphate of quinia is markedly beneficial, and that the use of cold baths, first advocated by the Germans, but now frequently employed in our larger hospitals for the reduction of the temperature in pneumonia, greatly lessens the severity of the disease and lowers the rate of mortality. The results thus obtained, we know, are not dependent upon any direct effect upon the pulmonary affection, but from a controlling influence over the pyrexia, just as in any essential fever. Therefore, would it not be well for us to take a retrogressive step and resume the term *febris pneumonica*, or pneumonic fever, which was used by Hoffmann nearly two hundred years ago ?

